# Management of Malignant Pericardial Effusion

**Published:** 2014-07-01

**Authors:** Mary Petrofsky

**Affiliations:** From Stanford Hospital and Clinics, Stanford, California

## ABSTRACT

**Case Study** 

In early 2010, Mrs. Y., a 58-year-old Japanese woman, underwent a routine mammogram that revealed left focal asymmetry. A biopsy demonstrated invasive ductal carcinoma, and she was referred to surgery. She underwent a lumpectomy and left axilla sentinel lymph node biopsy. Pathology confirmed an invasive ductal adenocarcinoma, moderately differentiated, 1.2 cm, estrogen receptor (ER)/progesterone receptor (PR) negative, and HER2/*neu* negative with negative surgical margins. Three left axillary sentinel lymph nodes showed no evidence of disease.

Mrs. Y. was diagnosed with stage I T1cN0Mx breast cancer. Her oncologist estimated that she had a 25% 10-year risk of relapse and a 13% 10-year risk of mortality, with an estimated 35% mortality benefit conferred by adjuvant therapy. Although it was recognized that triple-negative breast cancer confers a worse prognosis (Foulkes, Smith, & Reis-Filho, 2010), no available prognostic calculators considered hormone receptor status; the oncologist’s projections were overly optimistic. In a recent retrospective study in triple-negative breast cancer patients, (Hernandez-Aya et al. (2011) found a 5-year mortality rate of 16% for T1N0 patients and a 5-year relapse of 26% in these patients, similar to what Mrs. Y.’s oncologist had estimated for 10 years.

The oncologist recommended adjuvant therapy, and Mrs. Y. received four cycles of adjuvant docetaxel and cyclophosphamide followed by radiation therapy to the tumor bed and left breast. Her treatment followed the course recommended by the National Comprehensive Cancer Network Guidelines at that time (Carlson et al., 2009). She completed therapy in October 2010 without having experienced any dose reductions, delays, or complications.

Mrs. Y. continued to show no evidence of disease until early 2013, when she developed left neck and arm swelling. Computed tomography (CT) scan of the neck and thorax revealed a left supraclavicular mass compressing the left internal jugular and subclavian veins and enlarged cervical lymph nodes. A biopsy of the supraclavicular mass confirmed metastatic breast cancer. CT scan of the abdomen and pelvis showed no other sites of disease. She was treated with involved-field irradiation (16 Gy) to the sites of tumor recurrence and then was lost to follow-up.

Six months following completion of irradiation, Mrs. Y. presented to the emergency department (ED) with dyspnea. On review of systems, she reported a 3-month history of a progressive cough and a 3-week history of hoarseness with progressive shortness of breath (SOB) and difficulty speaking. Upon physical examination, her voice was hoarse, breath sounds were clear to auscultation, and there were palpable left supraclavicular nodes. She did not have muffled heart sounds, elevated jugular venous pressure (JVP), or pulsus paradoxus. In the ED, her vital signs were blood pressure (BP) 129/85, pulse 89, oxygen saturation 98%, and respirations 16. An electrocardiogram (ECG) revealed sinus rhythm with borderline T-wave abnormalities in diffuse leads and a heart rate of 94. Her troponin I level was zero. A CT angiogram revealed a moderate-sized pericardial effusion (Figure 1) and supraclavicular and mediastinal lymphadenopathy with no evidence of a pulmonary embolus. Ultrasound confirmed a moderate pericardial effusion with no evidence of tamponade.

**Figure 1:**
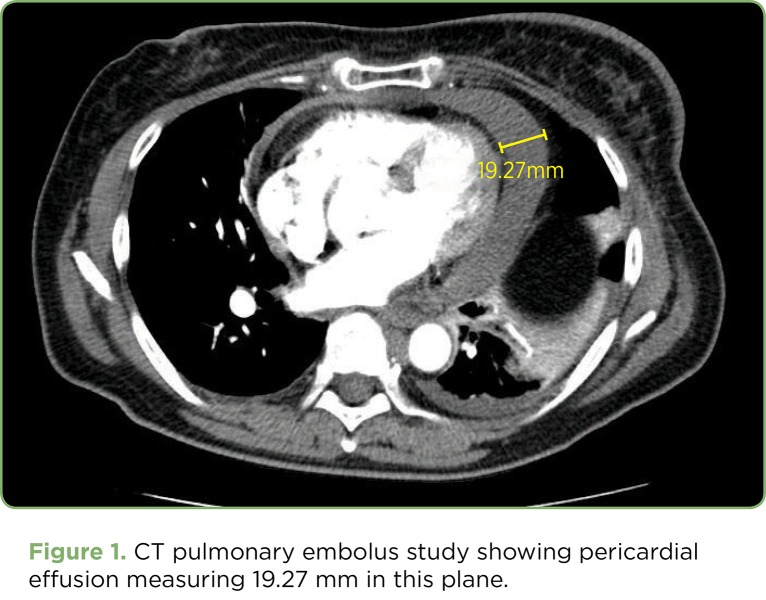
CT pulmonary embolus study showing pericardial effusion measuring 19.27 mm in this plane.

Overnight, Mrs. Y.’s SOB remained stable but her BP dropped to 70/49, which improved to 80/62 in response to a normal saline bolus. An ECG revealed sinus rhythm with partial resolution of the T-wave abnormalities and a heart rate of 65. Systolic blood pressure remained below 90 for several hours despite fluid boluses; by morning her BP had improved to 93/63. Echocardiography (echo) revealed a moderate-sized circumferential pericardial effusion with mild diastolic right atrium/right ventricle collapse, a dilated inferior vena cava, and mild mitral valve inflow variation with respiration, consistent with mild hemodynamic compromise. Left ventricle size and systolic function were normal.

Based on the echo findings, newly elevated JVP, and ongoing hypotension, Mrs. Y. was diagnosed with cardiac tamponade and suspected malignant pericardial effusion. Cardiology performed a diagnostic and therapeutic echo-guided pericardiocentesis and removed 260 mL of a bloody turbid fluid. A drain was left in, but it removed only 10 mL of fluid overnight and was removed the next day. Mrs. Y. had almost immediate relief of her dyspnea, and by day 3 she reported that she felt "a hundred times better" than she had at admission. She was discharged later that day after repeat echo showed a stable small pericardial effusion. Cytology of the pericardial fluid ultimately revealed metastatic adenocarcinoma consistent with the original breast cancer diagnosis.

## ARTICLE

The pericardium surrounds the heart and the great blood vessels and is composed of a thin visceral membrane, a fibrous parietal membrane, and the pericardial space between the membranes, which normally contains less than 50 mL of an ultrafiltrate of plasma known as pericardial fluid (Hoit, 2011; Braunwald, 2012). The parietal membrane is composed primarily of collagen and elastin fibers, which gives the membrane some elasticity (Braunwald, 2012). As a result of this elasticity, the normal pericardium has a nonlinear pressure-volume curve. Small pericardial fluid volume changes do not generally result in any change in pericardial pressure, but a large sudden increase in pericardial volume can cause a steep change in pericardial pressure, leading to tamponade (Imazio & Adler, 2013).

With a slowly enlarging pericardial effusion, the pericardial membranes stretch to accommodate the growing fluid volume without any significant change in the pericardial pressure until the limit of pericardial membrane stretch is reached. When pericardial fluid volume increases beyond the limit of membrane stretch, cardiac tamponade results (Imazio & Adler, 2013). Surprisingly, although the pericardium has many normal functions (Table 1 ), there are no significant consequences if the pericardium is removed or congenitally absent (Hoit, 2011; (Braunwald, 2012).

**Table 1:**
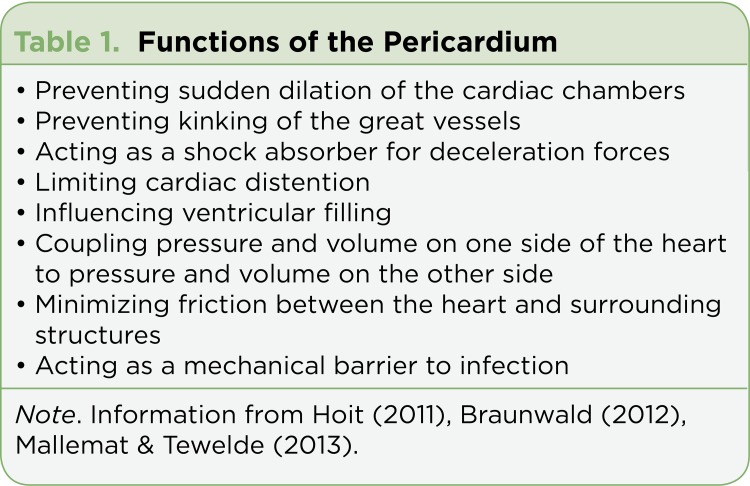
Functions of the Pericardium

## Etiology of Pericardial Effusions

Pericardial effusions can be idiopathic, infectious (most commonly viral), cardiac, autoimmune, medication-induced, radiation-induced, traumatic, metabolic, malignant, or the result of other causes (Imazio & Adler, 2013). The most common cause in cancer patients is a malignant effusion from lung or breast cancer (Lestuzzi, 2010; Pawlak-Cieślik et al., 2012), but nonmalignant etiologies (thoracic radiation, infection, autoimmune process, and medication) and other cancers (other solid tumors, hematologic malignancies, Hodgkin lymphoma, and non-Hodgkin lymphoma) can also cause effusions (Maisch, Ristić, & Pankuweit, 2010). Chemotherapeutic agents that can cause pericardial effusion include cyclophosphamide, cytarabine, dasatinib (Sprycel), doxorubicin, gemcitabine, and other cardiotoxic agents (Svoboda, 2010).

## Symptoms and Exam Findings

Effusions that develop quickly are the most likely to cause symptoms and physical exam findings (Seferović et al., 2013; Burazor, Imazio, Markel, & Adler, 2013). Dyspnea is the most common symptom in malignant pericardial effusion (Svoboda, 2010). Other symptoms include pleuritic chest discomfort, cough, fatigue, hoarseness from recurrent laryngeal nerve compression, and hiccups from phrenic nerve compression, with syncope being particularly concerning for tamponade (Borlaug, DeCamp, & Gangadharan, 2013; Refaat & Katz, 2011).

Pericardial effusions can be difficult to diagnose because clinical findings have poor sensitivity; tachycardia may be the only sign (Pawlak-Cieślik et al., 2012). The classic physical exam findings that are concerning for tamponade are collectively referred to as Beck’s triad: hypotension (often with a narrow pulse pressure), tachycardia, and muffled heart sounds. Beck’s triad was initially described for acute tamponade, which develops over minutes to hours; it is rarely seen in cancer patients with malignant pericardial effusions, who tend to develop subacute tamponade over days to weeks (Argulian, Herzog, Halpern, & Messerlin, 2012; (Borlaug et al., 2013; Imazio & Adler, 2013). Other signs of tamponade include elevated JVP and pulsus paradoxus (Refaat & Katz, 2011). Pulsus paradoxus above 10 mm Hg has been reported to be the most sensitive physical finding for tamponade but still only has a sensitivity of 82%, followed by tachycardia and elevated JVP, with sensitivities of 77% and 76%, respectively (Sherbino, 2009).

## Diagnostic Workup

**Figure 2:**
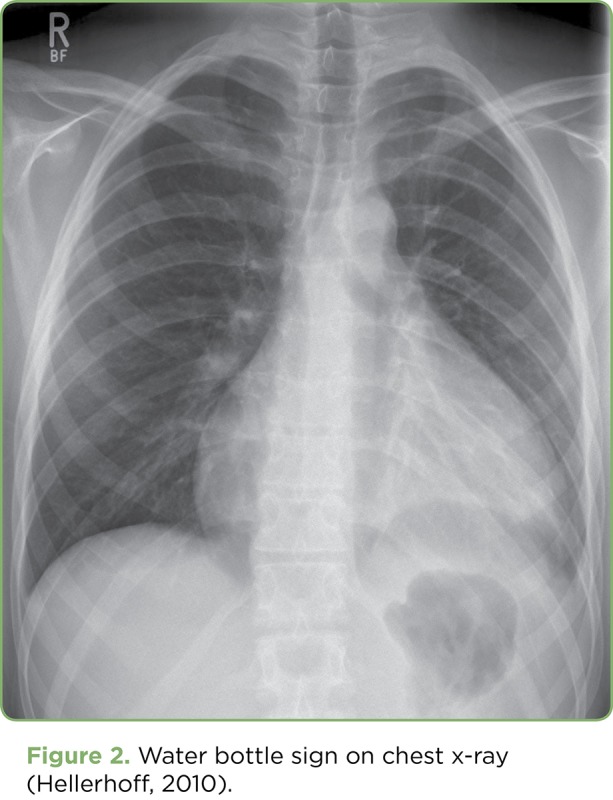
Water bottle sign on chest x-ray (Hellerhoff, 2010).

Given that dyspnea is the most common symptom, a chest x-ray is often the first study obtained. An enlarged cardiac silhouette with clear lungs (the "water bottle sign," as shown in Figure 2) is the classic finding in pericardial effusion, and concomitant pleural effusion is common in malignant pericardial effusions (Hoit, 2013a). The patient’s ECG may be normal, or it can demonstrate low QRS voltage, nonspecific ST- or T-wave changes, or electromechanical dissociation (agonal phase; Svoboda, 2010). Low QRS voltage is indicative of cardiac tamponade, but its absence does not rule out tamponade (Seferović et al., 2013). Low QRS voltage is most commonly associated with tamponade caused by a malignant pericardial effusion and usually resolves within a week of pericardiocentesis (Oliver et al., 2002). Elevated troponin I and creatine kinase myoglobin levels are commonly seen but appear to have no prognostic implication (Imazio et al., 2013).

Echo is the diagnostic standard, as it is the most useful imaging study for determining the presence, size, location, and hemodynamic effect of a pericardial effusion (Hoit, 2011). Although a CT of the thorax is generally not a good modality for determining the severity of an effusion, both CT and magnetic resonance imaging (MRI) may be superior to echo for determining the amount and distribution of pericardial fluid and whether an effusion is hemorrhagic or loculated (Bogaert & Francone, 2013). Cardiac chamber collapse on echo typically occurs before clinical hemodynamic failure (Mallemat & Tewelde, 2013). Echo findings consistent with cardiac tamponade include collapse of the right atrium at end diastole and the right ventricle in early diastole, reciprocal changes in left and right ventricular volumes with respiration, increased respiratory variation of mitral and tricuspid valve inflow velocities, and inferior vena cava (IVC) dilatation with less than a 50% reduction in IVC diameter during inspiration (Hoit, 2013b). See Table 2 for a list of key elements in diagnosing malignant pericardial effusion.

**Table 2:**
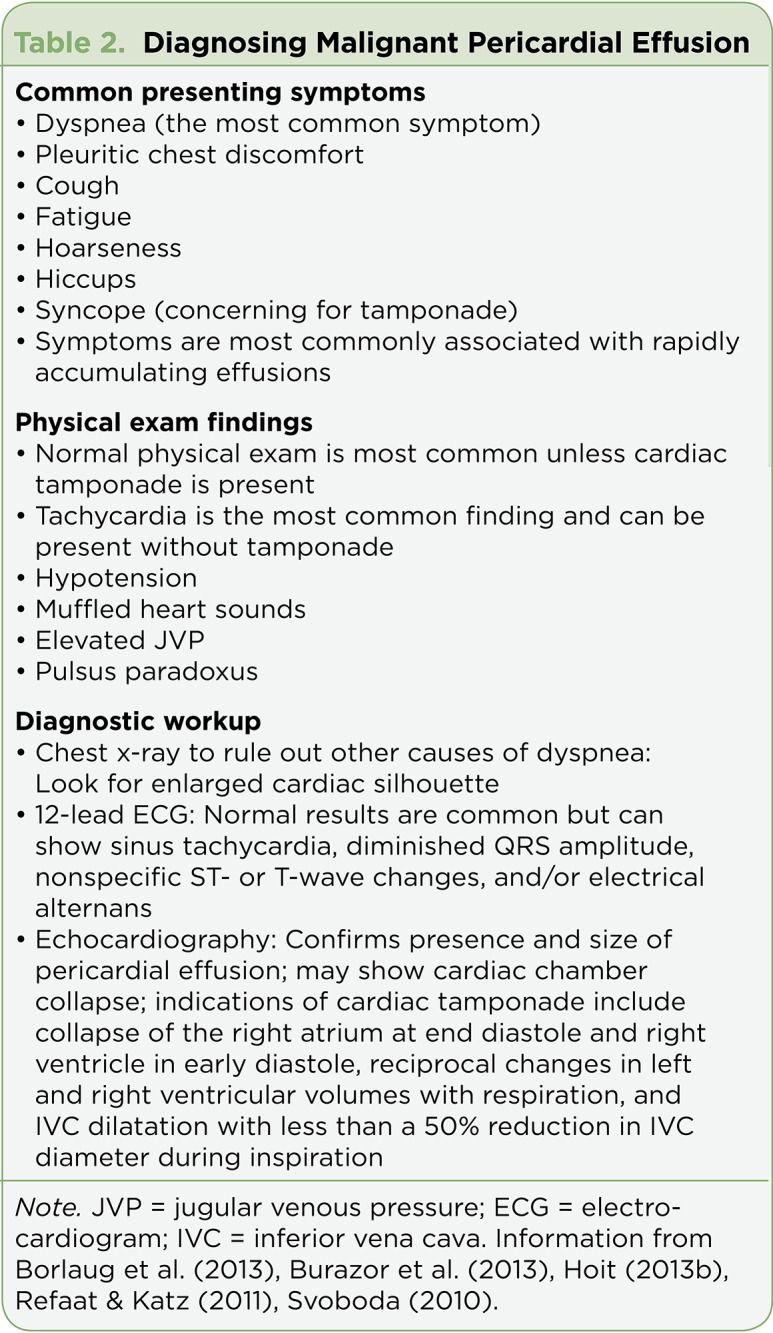
Diagnosing Malignant Pericardial Effusion

## Initial Management

In 2004, the Task Force on the Diagnosis and Management of Pericardial Diseases of the European Society of Cardiology published guidelines for the management of pericardial effusion (Maisch et al., 2004). Although there are many articles that specifically address pericardial effusions in patients with cancer, there have been no randomized controlled trials or prospective intervention trials (Burazor et al., 2013). Treatment of pericardial effusion secondary to malignant disease requires consideration of the patient’s prognosis from the underlying malignancy, the availability of local expertise, and the cardiovascular and medical status of the patient (Imazio & Adler, 2013). Stable patients without evidence of tamponade can be managed with careful monitoring, serial echo studies, avoidance of volume depletion, and therapy aimed at the underlying cause of the pericardial effusion (Borlaug et al., 2013), regardless of effusion size (Mallemat & Tewelde, 2013).

Patients with evidence of tamponade who are hypovolemic should be given volume resuscitation if systolic BP is below 100 mmHg (Sagristà-Sauleda, Angel, Sambola, & Permanyer-Miralda, 2008). In tamponade, there is a significant increase in the pericardial pressure, and the central venous pressure must be kept higher than the pericardial pressure in order for the heart to fill. If volume resuscitation results in hemodynamic improvement, such patients may be observed closely without urgent need for pericardiocentesis (Hoit, 2013b). In patients with cancer, pericardiocentesis is indicated for symptomatic cardiac tamponade and for high suspicion of tuberculous or infectious etiology (Maisch et al., 2013; Sagristà-Sauleda et al., 2011).

In general, pericardial effusion drainage should be considered if the echo demonstrates chamber collapse and the patient is symptomatic; drainage is not necessarily indicated for echocardiographic right atrial collapse in an asymptomatic patient (Hoit, 2011; Maisch, Ristić, Seferovic, & Tsang, 2011). Furthermore, pericardiocentesis is associated with risk and will not always resolve symptoms (Mallemat & Tewelde, 2013). Major complications of pericardiocentesis in a large study (1,127 procedures) occurred in 1.2% of echo-guided cases and included heart chamber laceration requiring surgery, pneumothorax, ventricular tachycardia, and bacteremia (Tsang et al., 2002). Minor complications requiring monitoring but no intervention occurred in 3.5% of echo-guided cases and included transient heart chamber entrance and small pneumothorax (Tsang et al., 2002).

If pericardiocentesis is required, different approaches can be used (Table 3). Pericardiocentesis may be followed by catheter placement for ongoing fluid removal until the rate of fluid return is less than 20 to 30 mL over 24 hours (Imazio, Spodick, Brucato, Tinchero, & Adler, 2010). The risk of recurrence of pericardial effusion is significantly reduced when pericardiocentesis is followed by extended catheter drainage, with 6-month recurrence rates of 14% vs. 27% with and without extended drainage (Tsang et al., 2002). However, it is important to note that only 33% of the sample in this study had a malignant pericardial effusion, and malignancy was independently correlated with increased risk of effusion recurrence (Tsang et al., 2002). Repeat echo should be performed after pericardiocentesis to confirm adequate fluid removal and to detect early recurrent fluid accumulation (Cheitlin et al., 2003).

**Table 3:**
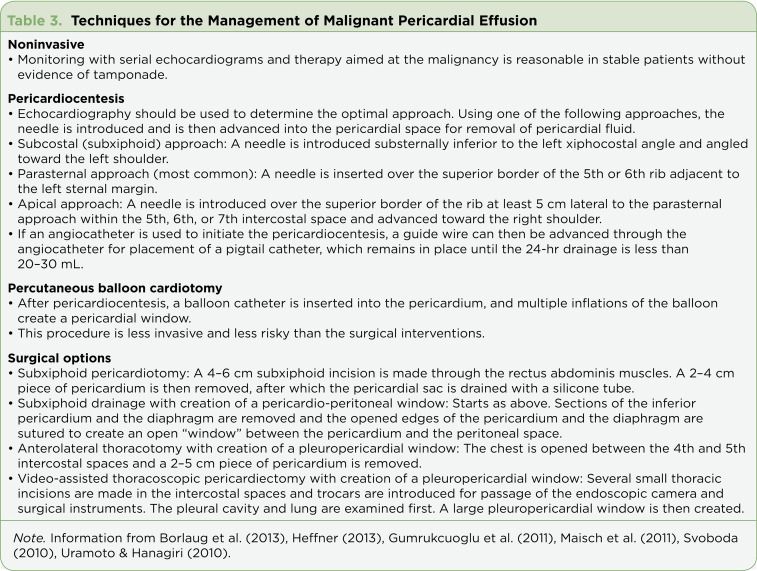
Techniques for the Management of Malignant Pericardial Effusion

The most helpful pericardial fluid studies are cytology, Gram stain, and bacterial/fungal cultures, although negative cytology is not sufficient to exclude malignancy (Maisch et al., 2011). In immunocompromised patients, polymerase chain reaction studies for viruses can be helpful, such as cytomegalovirus in transplant patients (Maisch et al., 2011). Protein, lactate dehydrogenase, glucose, and cell count have not been shown to be diagnostically helpful because they do not reliably distinguish malignant from benign pericardial effusions (Karatolios, Pankuweit, & Maisch, 2013).

There is significant controversy regarding the best time for consideration of surgical rather than percutaneous decompression of pericardial effusions in patients with malignancy. It is well documented that pericardial effusions have a higher risk of recurrence after pericardiocentesis compared with surgical interventions, with recurrence rates as high as 90% in patients with malignancy (Refaat & Katz, 2011). Although surgical interventions result in increased discomfort and morbidity compared with pericardiocentesis (Svoboda, 2010), for a patient who experiences multiple symptomatic recurrences of a malignant pericardial effusion, a surgical decompression may result in overall improvement in quality of life with more time outside the hospital despite the initial increase in morbidity. A variety of surgical options exist, some of which can create a pericardial window to allow ongoing drainage to the pleural or peritoneal space (Table 3).

## Prevention of Recurrence

The guidelines of the European Society of Cardiology include the following options to prevent recurrence of malignant pericardial effusions: systemic antineoplastic treatment, intrapericardial instillation of sclerosing or cytotoxic agents, percutaneous balloon pericardiotomy, surgical subxiphoid pericardiotomy or pleuropericardiotomy, and radiation therapy (Maisch et al., 2004). Although anti-inflammatory medications have been shown to be very useful in inflammatory pericardial effusions, they have little utility in malignant effusions (Imazio & Adler, 2013). The standard recommendation is systemic chemotherapy to control the cause of the malignant effusion (Lestuzzi, 2010; Maisch et al., 2004; Refaat & Katz, 2011). Instillation of sclerosing agents has been shown to have little impact on effusion reaccumulation or survival (Kunitoh et al., 2009). Percutaneous balloon cardiotomy is less invasive than surgical interventions and has a good success rate (Maisch et al., 2011). The various surgical options outlined in Table 3 have a lower rate of effusion recurrence than do percutaneous interventions, but they incur higher morbidity. Radiation therapy was used historically to control malignant pericardial disease (Cham, Freiman, Carstens, & Chu, 1975), but it is no longer favored, as it can also cause pericardial effusions (Svoboda, 2010).

Intrapericardial instillation of cisplatin is favored in some centers, with a reported response rate of 93%, a response duration of 3 months, and greater efficacy in lung cancer vs. breast cancer (Maisch et al., 2010). Intrapericardial instillation of thiotepa has been used in breast cancer with good effect (Burazor et al., 2013). Although a retrospective review showed significant improvement in outcomes for patients treated with both intrapericardial and systemic chemotherapy (Lestuzzi et al., 2011), the use of such practices has not been widely adopted due to the potential for pain caused by introduction of the agents and concern for later development of constrictive pericarditis (Borlaug et al., 2013).

## Prognostic Implications

Development of a symptomatic pericardial effusion in a patient with a malignancy confers a poor prognosis, with a median survival time of 2 to 5 months from the time of detection (Dequanter, Lothaire, Berghmans, & Sculier, 2008;). Prognosis may be slightly better in the subset of patients with negative cytology (Neragi-Miandoab et al., 2008), hematologic rather than solid malignancies (Svoboda, 2010), and breast rather than lung or other solid tumors (Kim et al., 2010). Leukemic pericardial effusions have been shown to be relatively frequent (20%) but generally asymptomatic and small, with a large retrospective review showing that only 3% of such pericardial effusions required intervention (Sampat et al., 2010).

## Case Discussion and Update

**Figure 3:**
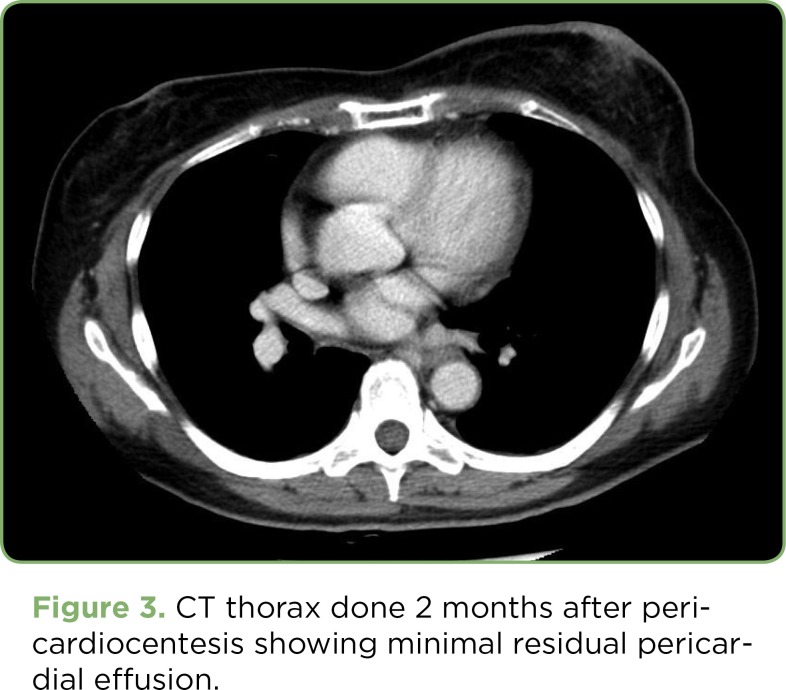
CT thorax done 2 months after peri - cardiocentesis showing minimal residual pericar - dial effusion.

After the pericardiocentesis, Mrs. Y. began systemic chemotherapy as recommended. She had no further issues related to the pericardial effusion in the 5 months after the pericardiocentesis, although her metastatic breast cancer continued to progress. Repeat CT imaging showed only a small residual effusion (Figure 3).

Mrs. Y.’s case is somewhat unusual in that she became symptomatic with a relatively small effusion. In slowly accumulating effusions (the typical pattern in malignancy), patients are rarely symptomatic with an effusion size less than 500 mL (Schoen & Mitchell, 2010) and may remain asymptomatic until 2,000 mL or more has accumulated (Hoit, 2013a). However, symptoms are a result not only of effusion size, but also of the rate of fluid accumulation relative to pericardial stretch and how effectively the heart compensates for the reduced heart chamber size (Saito et al., 2008).

Mrs. Y.’s symptoms were typical, as she presented with shortness of breath, the most common symptom, and subsequently developed hypotension. Her rapid improvement with pericardiocentesis is also typical although not universal. Mrs. Y. had multiple risk factors for pericardial effusion, including metastatic breast cancer, history of thoracic radiation, and treatment with cyclophosphamide. Given that these risk factors are present for the majority of patients with metastatic breast cancer, it is important for advanced practitioners to have a high index of suspicion for pericardial effusion in addition to malignant pleural effusion and pulmonary embolus when such patients present with shortness of breath.

## Considerations for Advanced Practitioners

Pericardial effusion should be suspected in any patient with a malignancy and any of the following symptoms: dyspnea or pleuritic chest pain, new radiographic cardiomegaly without pulmonary congestion, unexplained persistent fever, presence of an isolated left pleural effusion, or hemodynamic deterioration of unknown etiology (Hoit, 2013b). The most common malignancies causing pericardial effusion are breast and lung cancer, followed by Hodgkin lymphoma, non-Hodgkin lymphoma, and leukemia (Lestuzzi, 2010). Consequently, it is especially important for advanced practitioners to have a high index of suspicion in a patient with one of these malignancies presenting with shortness of breath. Radiation-induced pericardial effusion may occur during the radiation therapy itself or up to 20 years after therapy (Lee, Finch, & Mahmud, 2013), and a history of thoracic radiation therapy should further increase the suspicion of pericardial effusion.

A patient with a moderate pericardial effusion may be minimally symptomatic and may have no specific physical exam findings and a normal ECG. If a pericardial effusion is within the differential diagnosis for the reasons outlined here, echocardiography is the most specific and clinically important diagnostic tool, as it can provide evidence of cardiac compromise prior to the development of overt tamponade. In pericardial effusions that develop slowly and do not cause hemodynamic compromise, systemic chemotherapy is often a better option than invasive intervention. Any patient who develops symptomatic tamponade from a malignant pericardial effusion needs intervention for the effusion, preferably with pericardiocentesis followed by systemic chemotherapy, if these interventions are aligned with the patient’s goals of care. For recurrent symptomatic malignant pericardial effusions, a surgical intervention may, after the initial postsurgical recovery period, improve quality of life and reduce hospital stays. 
